# Customizable Presentation Attack Detection for Improved Resilience of Biometric Applications Using Near-Infrared Skin Detection

**DOI:** 10.3390/s24082389

**Published:** 2024-04-09

**Authors:** Tobias Scheer, Markus Rohde, Ralph Breithaupt, Norbert Jung, Robert Lange

**Affiliations:** 1 Institute of Safety and Security Research, University of Applied Sciences Bonn-Rhein-Sieg, Grantham-Allee 20, 53757 Sankt Augustin, Germany; markus.rohde@h-brs.de (M.R.); norbert.jung@h-brs.de (N.J.); robert.lange@h-brs.de (R.L.); 2 Federal Office for Information Security, Godesberger Allee 185-189, 53175 Bonn, Germany; ralph.breithaupt@bsi.bund.de

**Keywords:** presentation attack detection (PAD), sensor resilience, biometrics, skin detection, optical sensor, spectroscopy, near infrared, analog/digital signal processing

## Abstract

Due to their user-friendliness and reliability, biometric systems have taken a central role in everyday digital identity management for all kinds of private, financial and governmental applications with increasing security requirements. A central security aspect of unsupervised biometric authentication systems is the presentation attack detection (PAD) mechanism, which defines the robustness to fake or altered biometric features. Artifacts like photos, artificial fingers, face masks and fake iris contact lenses are a general security threat for all biometric modalities. The Biometric Evaluation Center of the Institute of Safety and Security Research (ISF) at the University of Applied Sciences Bonn-Rhein-Sieg has specialized in the development of a near-infrared (NIR)-based contact-less detection technology that can distinguish between human skin and most artifact materials. This technology is highly adaptable and has already been successfully integrated into fingerprint scanners, face recognition devices and hand vein scanners. In this work, we introduce a cutting-edge, miniaturized near-infrared presentation attack detection (NIR-PAD) device. It includes an innovative signal processing chain and an integrated distance measurement feature to boost both reliability and resilience. We detail the device’s modular configuration and conceptual decisions, highlighting its suitability as a versatile platform for sensor fusion and seamless integration into future biometric systems. This paper elucidates the technological foundations and conceptual framework of the NIR-PAD reference platform, alongside an exploration of its potential applications and prospective enhancements.

## 1. Introduction

Biometric solutions are used more and more frequently for the authentication of individuals. Therefore, the use of biometric counterfeits to overcome such systems poses a serious security problem. The detection of such attacks is called presentation attack detection (PAD) and is generally considered unsolved. Most biometric systems use additional sensors and machine-learning-based methods as a PAD approach and work reasonably well. Unfortunately, this approach can only detect previously learned situations, which can lead to security risks when new, unknown attacks are carried out. A solution to this problem for optical biometric sensors could be the reliable detection of skin in the near-infrared (NIR) remission spectrum, since a physical property is observed. In addition, the signal processing of this technology is easy to understand, making it less error-prone and more resilient than other methods. This paper is organized as follows: a method of reliable skin detection independent of skin type is presented, followed by related work, a description of the sensor working principle, dedicated hard- and software design and operation considerations, the first experimental results and an outlook. The main contributions are as follows:

- An introduction to the concepts behind reliable skin detection in the NIR remission spectrum;
- The technological background of a multispectral sensor system that utilizes a lock-in amplifier approach and is well suited for PAD in many biometric applications;
- A modular development and testing platform for further research and evaluation activities.

### 1.1. Near-Infrared Skin Detection

**Figure 1:**
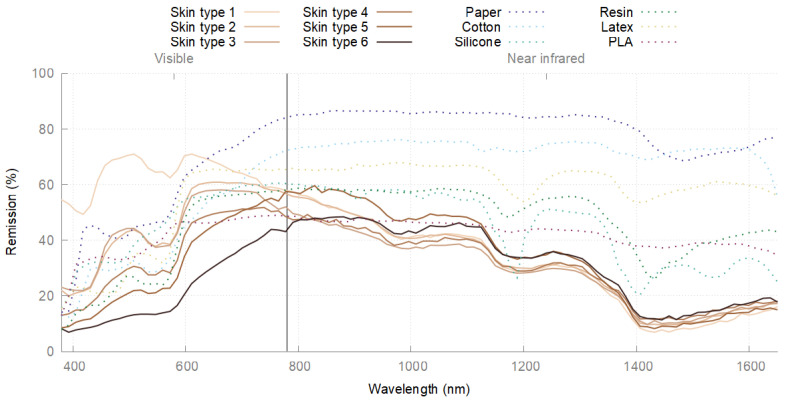
Remission spectra of human skin and exemplary artifact materials.

The spectral remission properties of human skin in the NIR have been known for a long time. Studies of these particular properties date back as early as the fifties of the twentieth century. Jacquez et al. showed that spectral remission above a wavelength of 1.2 μm is largely independent of skin type or melanin content and essentially depends on the high water content of human skin [1]. Spectroscopy can be used to illustrate this fact. Figure 1 shows the spectral remission curves of the six different skin types according to Fitzpatrick [2]. While the curves diverge widely in the visible range, they converge above approximately 900 nm and describe a uniform pattern. In addition to the different skin types, exemplary artifact materials are shown, which are clearly different from skin in the NIR spectral range and can thus be differentiated very well.

Using a spectrometer has some significant disadvantages for the real applications considered here. Typically, a controlled environment is mandatory, i.e., no ambient light and a defined measurement geometry. Also, the acquisition speed is rather slow, and the costs are quite high. Therefore, most sensor systems sample the near-infrared spectrum only at a few narrow supporting points for their surface material analysis. Most known skin detection sensor systems have a working range between 1000 nm and 1600 nm, which applies well to the spectral sensitivity of Indium-Gallium-Arsenide-based detectors (InGaAs). Since biometric consumer-grade sensors use silicon detectors with sufficient sensitivity only below 1000 nm, their coexistence with near-infrared skin detection is possible without mutual interference.

### 1.2. Related Work

Taking advantage of the remission properties of human skin in the near-infrared is not a new idea. As early as 1985, a sensor for skin recognition was described in a patent by Hacskaylo [3]. The sensor uses a broadband light source and a total of three detectors with different bandpass filters at 1200 nm, 1500 nm and 1720 nm. The remission intensities generated at the three wavelengths are compared with each other to identify the spectral signature of skin.

In 1999, Pavlidiz et al. [4] proposed a dual-band camera system consisting of two co-registered cameras with rather wide passbands, one at 1100–1400 nm and the other at 1400–1700 nm. The system was developed for the automatic detection and counting of vehicle occupants to enable the control of the use of lanes reserved specifically for carpooling. The two cameras produce images of the same scene, and the binary skin information is formed by the weighted difference between the two images, followed by a threshold. Since the system should work with artificial as well as natural lighting, the weighting must be adapted to the spectral distribution of ambient lighting. A patent for this process was granted in 2002 [5].

At the U.S. Air Force Institute of Technology, Nunez and Mendenhall [6,7] researched the use of hyperspectral imagery for skin detection with the aim to find missing people in aerial rescue missions. The hyperspectral images contained 81 spectral bands ranging from 900 nm to 1744 nm, and the normalized difference of two wavelengths (1100 nm and 1400 nm) was applied to detect skin. Furthermore, they described a physical model of human skin and proposed a multicamera system with narrow bandpass filters on each camera for field application.

The systems considered so far are highly dependent on the spectral distribution of ambient light due to their passive-filter-based design. In contrast to this, the Institute for Safety and Security Research at Bonn-Rhine-Sieg University of Applied Science has been working on skin detection systems with active lighting for many years. All of our sensors have narrowband LED-based lighting and a wideband InGaAs-based detector in common, which makes them widely independent of ambient light. Schwaneberg et al. [8,9,10] developed point sensors for safety applications to safeguard dangerous machines or areas. Examples include a safety feature on a drilling machine and a guard on a table saw (a mechanism blocks the saw blade when skin is near the cutting area). Steiner et al. [11,12] transferred the concept to an imaging system by employing a dedicated camera with an InGaAs-based sensor instead of single photodiodes. The proposed system is able to combine face biometrics and PAD with the same detector. Unfortunately, the used InGaAs camera suffers from low spatial resolution (e.g., VGA standard) and high costs due to their complex construction compared to consumer-grade silicon detectors. A patent for using active near-infrared lighting in biometric applications was granted in 2011 [13].

The previously introduced active sensor systems acquired the remission information for each wavelength sequentially, meaning that only one LED type (or wavelength) is active at one time. This measurement method can be applied to much slower detectors, like cameras, but suffers in some relevant practical applications (e.g., high ambient light, fast movements or other active sensor systems). Therefore, in the last several years, we established another well-studied and employed measurement method (lock-in amplifier) in our near-infrared sensors [14]. By modulating the different LED types with different frequencies, all remission information can be acquired simultaneously, and the signal quality can be massively improved. A conceptually similar system was presented by Hintenaus and Trinker [15], where three near-infrared wavelengths (1300 nm, 1450 nm and 1650 nm) were used to measure the moisture content in paper.

To the best of our knowledge, there is no published work that utilizes a lock-in amplifier approach for a near-infrared skin detection system.

### 1.3. Aim of This Work

This paper presents a new measurement methodology for near-infrared skin detection systems that has not yet been published in this form. In addition to near-infrared skin detection, an accurate distance measurement is integrated, as this combination is particularly relevant for PAD in a variety of biometric contexts. The proposed approach includes significant system miniaturization, the development of a new signal processing chain based on (digital) lock-in amplifiers, and the incorporation of time-of-flight (ToF) for distance measurement. These advancements, while innovative, play a crucial role in enhancing the scope and applicability of the technology in biometric applications.

A practical development example will be used to highlight the most critical technical challenges. The sensor system proposed has the potential to serve as a modular development and testing platform for future research. It allows the separate optimization of all elements, like NIR lighting, modulation settings, filter parameters, timing constraints and signal analysis. The following sections provide an overview of the key hardware and software components and the underlying design considerations. Finally, we present examples of the material analysis capabilities of this new approach.

## 2. Sensor Working Principle

**Figure 2:**
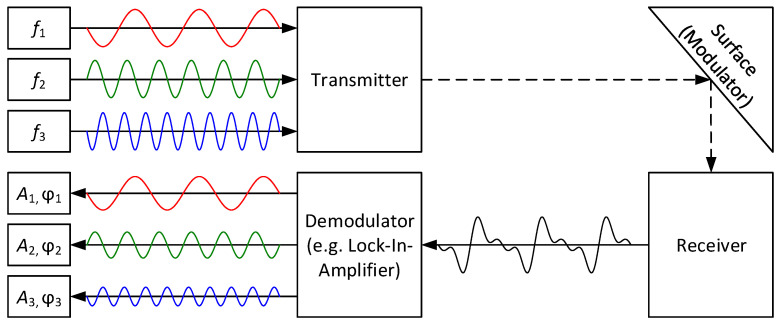
Multispectral sensor working principle.

The proposed multispectral sensor system employs a multichannel lock-in amplifier approach, which is illustrated in Figure 2 (amplitude modulation with frequency multiplexing). In this approach, carrier signals with known frequencies $f_{i}$ are emitted by a transmitter and are reflected on a measurement surface. A receiver then receives the superimposed and attenuated signals to calculate their amplitude $A_{i}$ (and phase shift $\varphi_{i}$) with a suitable demodulator (e.g., lock-in amplifier). Since the remission intensity is dependent on the wavelength and observed material, the targeted information is represented by the received carrier signal amplitudes.

**Figure 3:**
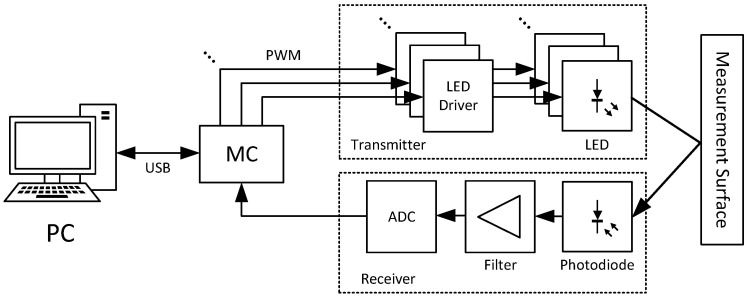
Simplified multispectral sensor implementation schema.

In modern sensor systems of this type, demodulation is often completely digital, since modern microcontrollers usually have sufficient computing power. This enables cost-efficient implementations with far fewer (expensive) analog components, while still reaching a high level of signal quality. The simplified implementation schema of the proposed sensor is shown in Figure 3. There, a suitable microcontroller is responsible for generating the different carrier signals for driving the transmitting side, as well as reading the measurement data on the receiving side.

It is common to use square waves as carrier signals instead of sine waves, since the generation of the former is often much simpler, and the disadvantages can be mitigated by system design. The odd harmonics of a square wave can be attenuated by a suitable low-pass filter (anti-aliasing), and interference between the different carrier signals can be avoided by choosing suitable modulation frequencies. Modern microcontrollers usually have a sufficient number of built-in timers that can be used to create many independent square waves directly in hardware by generating pulse-width modulation (PWM) signals with a 50% duty cycle. The PWM signals are then used to control the LED drivers, which are special integrated circuits that drive the LEDs at a constant, preset current with high accuracy.

A photodiode then receives the superimposed and reflected signals, and an analog filter prepares them for digitization with an analog-to-digital converter (ADC). After digitizing the signal, further processing is carried out entirely in software with a digital lock-in amplifier approach. In our proposed sensor system, a connected PC can retrieve the measurement data for further analysis. Since complete digital signal processing can also be performed directly by the microcontroller, the PC is not required for the operation. Instead, it can be replaced by a suitable actuator to output the results.

During operation, the transmitter and receiver do not need to be synchronized. The unknown phase shift between carrier signals and the detector can be handled by a suitable demodulation algorithm, e.g., a dual-phase lock-in amplifier or Fast Fourier Transform (FFT). Both actions could even be performed with separate microcontrollers, but then precise clocks are recommended to avoid leakage due to phase drift between the two parts.

It should be noted that the sensor schema described here does not include details on the optical components for simplicity. Depending on the application, suitable optical components are required in front of the LEDs (e.g., for beam shaping) and the photodiodes (e.g., to limit the measuring spot size).

In the following sections, some parts of the described sensor schema with major relevance are explained in more detail.

## 3. Hardware Design Considerations

The sensor system should be a mobile test platform and, therefore, as simple and versatile as possible. For this reason, it should have compact dimensions and not require an additional external (laboratory) power supply. Thus, a single USB port is used for both communication and the supply voltage. Since it is a small USB device, it can be easily transported and used on a notebook directly in the field for experiments or even integrated into other systems. Another important requirement is that no expensive custom-made (optoelectronic) parts be used. By using standard (off-the-shelf) components, it is shown that this sensor technology can also be used in applications where cost is an important consideration. As mentioned above, the receiver must be based on InGaAs, and the transmitter should cover most of the spectral range of the detector.

### 3.1. General Electronic Design

**Figure 4:**
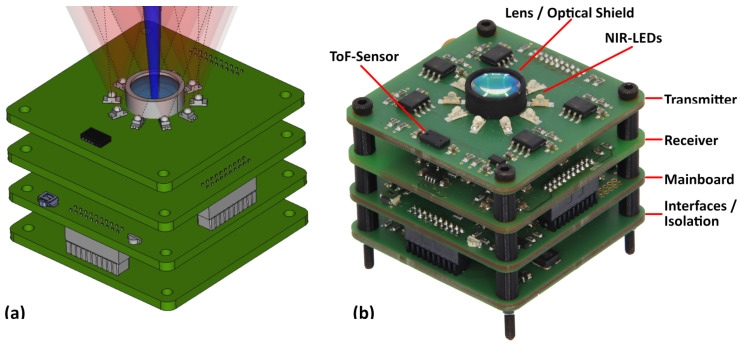
Images of the PCB stack: (**a**) CAD. (**b**) Photo.

For easy development and replacement, the sensor consists of stacked printed circuit boards (PCBs), as shown in Figure 4. This way, individual parts can be replaced, and different hardware variants can be easily tested. All PCBs are assembled on only one side to allow easy reflow soldering in our own laboratory (hand-made prototypes). The four boards of the stack contain the following components:

**Interface Board:**
  External interfaces (USB, JTAG), the main voltage regulation (including power monitoring) and an isolation. The isolation should allow operation even with noisy USB voltage supplies. An additional power monitor is used to monitor the supply voltage and power consumption to ensure safe operation.
**Mainboard:**
  A microcontroller (Cortex M7), a button, an RGB-LED and an expansion header for further use. The button and RGB-LED are intended for standalone operation without a PC. An expansion header provides interrupt IOs and SPI and can be used for later integration into other systems. The mentioned tasks require a microcontroller with high performance (standalone operation) and high connectivity (five communication buses, eight PWM signals and several interrupt IOs). For easier routing, the connection ports for the receiver and the transmitter are kept separate.
**Receiver Board:**
  A photodiode, an analog filter circuit and an ADC. More detailed information about the receiver components is covered in Section 3.2 below. Since this layer tunnels the connection to the upper transmitter board, special care must be taken to avoid interference with the analog circuitry. The high LED current and the high-frequency PWM signals can generate significant electromagnetic noise, so sufficient measures must be taken in the PCB layout. Extensive noise levels can severely limit the sensor range, even with sophisticated calibration methods. The receiver port consists of several trigger IOs and an SPI interface with over 50 MHz for sampling 16-bit values with up to 1 Msps.
**Transmitter Board:**
  An LED driver, NIR-LEDs and a ToF (time-of-flight) sensor. More detailed information about the transmitter components is discussed in Section 3.3. The transmitter port consists of five independent PWM signals and an I2C interface connecting an additional distance sensor.

### 3.2. Receiver

The detectors used must have sufficient spectral sensitivity for the used transmitter wavelengths and be fast enough for the modulation frequencies. Typical InGaAs photodiodes have a spectral sensitivity of 900 nm to 1700 nm and a typical diameter of the photosensitive area of about 300 μm. Larger sizes usually result in slower responses, but this size is more than fast enough for the targeted carrier signal frequency range of 10 kHz to 20 kHz.

Two different receiver variants with varying photodiodes were prepared. In order to achieve a more focused measurement with an external lens (9 mm focal length), one receiver has a photodiode with an integrated lens (viewing angle approx. 20°) and the other does not. The integrated lens variant has a relatively large measurement spot (>5 cm at 30 cm distance), produces a strong signal and has sufficient precision for close-range applications (up to 20 cm). The focused variant has a more precise measurement spot (about 1 cm at 30 cm distance) and is capable of detecting a single finger at a greater distance.

When developing optical components for multispectral sensor systems, the different focal lengths due to chromatic aberration must be taken into account. Since the focal length provided by the lens manufacturer applies to the visible range (typically about 550 nm), a slight shift in the lens position is necessary to adjust to the (slightly longer) focal length in the near-infrared. The exact displacement distance depends on the focal length of the lens, the wavelengths used and the measurement distance and can be determined using optics simulations.

**Figure 5:**
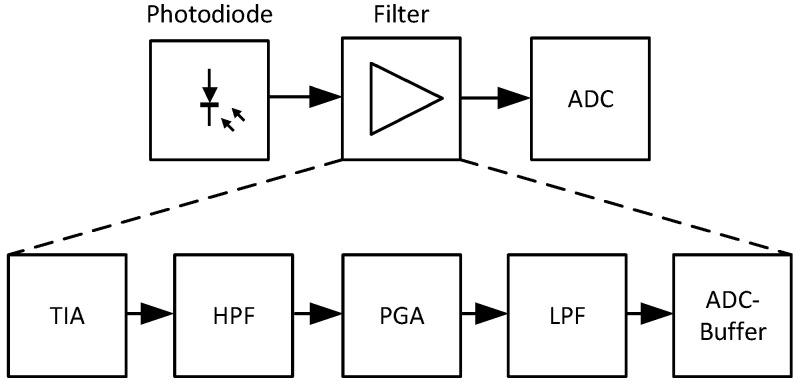
Analog filter chain.

The analog filter prepares the photodiode signal (photo-current) for later quantization with an ADC (voltage-based). Typical filter blocks are illustrated in Figure 5, and, depending on the application, more or less effort can be expended, and more elaborate active filters or simple passive filters can be used. The different filter blocks have the following tasks:

**Transimpedance Amplifier (TIA):**
  The mandatory transimpedance amplifier converts the photo-current of the photodiode into a voltage for further processing. Since high gains (≥$10^{6}$) and feedback capacitors (for stability) are usually required, a fast operational amplifier with a high gain–bandwidth product (GBWP) must be used. The feedback components (resistor and capacitor) introduce an additional low-pass filter, and a high GBWP allows for a lower capacitance while still remaining stable. This enables higher amplifications before the cut-off frequency reaches the targeted signal frequency range.
**High-Pass Filter (HPF):**
  With a high-pass filter, all static signal components (e.g., ambient lighting) and low-frequency noise (e.g., power hum) can be removed from the signal. This should be carried out early, before further signal amplification. In a closed/controlled environment with no external lighting, this filter could potentially be omitted. Implementation could be realized with active components (more sophisticated) or as a simple passive (low-cost) solution.
**Programmable-Gain Amplifier (PGA):**
  An additional gain step is highly recommended. In addition to a fixed amplification stage (e.g., 10×–100×), a programmable amplifier (e.g., 1×–64×) can be useful. The signal intensity decreases with the square of the measurement distance (surface of a sphere), and different materials can behave very variably in reflectance. In addition, skin typically has a rather high absorbance in the NIR (increasing with ascending wavelength) in comparison to many other materials. With a variable gain step, some of these effects could be mitigated, and a more stable operation with greater working ranges could be achieved, since the measurement distances and materials (low or high reflectance) are usually unknown.
**Low-Pass Filter (LPF):**
  The main task of the low-pass filter is to attenuate all unwanted high-frequency signal components to avoid aliasing in the sample data (anti-aliasing filter). This is why the cut-off frequency highly depends on the transmitter frequencies and the ADC sample rate. Typically, the cut-off frequency should be set as low as possible and the sample rate as high as possible to allow for a higher degree of oversampling. With oversampling, low-pass filtering can be performed with a lower-order filter while still achieving a good signal quality.
**ADC Frontend:**
  The ADC frontend depends highly on the employed ADC, and for low-cost solutions (12 bits and lower), this step can often be omitted. For higher-performance ADCs (16 bits+), the manufacturer’s recommendations should be considered. Often, an additional buffer (special high-GBPW amplifier) or other circuits (e.g., balancing flyback circuit) are needed to achieve the promised characteristics.

The final step in the receiver chain is an ADC, which digitizes the filtered analog signal for further digital processing. For low-cost solutions (12 bits and lower), the controller’s internal ADCs are often sufficient. It is also a cost-effective way to obtain many input channels for multiple measurement points, since some consumer controllers have multiple internal high-speed ADCs (>2 MSps), each with several channels. However, the proposed sensor hosts the receiver (including the ADC) on an external PCB for easy interchangeability. To achieve high amplitude resolution and signal quality, a high-performance ADC with a nominal resolution of 16 bits and a sample rate up to 1 Msps was chosen (AD4004).

Other important components of the receiver are the analog supply and reference voltages. For high performance, a separate voltage regulation for the analog circuit part is recommended to prevent any interference from the high-frequency digital circuits. In systems with a single supply voltage (e.g., USB), virtual ground references can be used to avoid the need for negative supply voltages. For this purpose, a reference voltage with dual balanced output ($V_{ref}$ and $V_{ref}/2$) is used to generate the required voltages for the ADC (reference voltage $V_{ref}$) and the analog filter chain (virtual ground $V_{ref}/2$). This way, the signal can oscillate around half of the reference voltage (virtual ground), and the ADC input range can be fully utilized. Note that some amplifiers (e.g., high-performance ADC buffers) do not typically operate rail-to-rail, so a voltage margin must be planned for.

### 3.3. Transmitter

Suitable LEDs must be chosen to cover most of the InGaAs detection range to enable a wide range of experiments. Since bare LED diodes emit in almost all directions, some kind of beam-forming is usually required. Some standard LED packages include integrated lenses to focus outgoing light, and for the targeted spectral range, there are typically two LED packages with lenses available (“off-the-shelf”): a surface-mounted device package (SMD) type 1206 and a through-hole variant (5 mm package). The SMD variants are smaller but have a rather wide viewing angle of 40°, making them suitable for use in close-range and small-dimension systems. In contrast, the through-hole variants have a smaller viewing angle of 20° and more radiant power (can withstand more current), which makes them more suited for long-range applications. For our proposed sensor system, a total of five SMD-LED types with integrated lenses were chosen, described in the Table 1 below.

Common to these LED types is that as the wavelength ($\lambda_{p}$) increases, the FWHM (full width at half maximum) or spectral bandwidth ($\Delta \lambda_{0.5}$) increases, and the radiant power ($\Phi_{e}$) decreases. The sensor employs two LEDs per wavelength and arranges them in a ring light around the receiver aperture on opposing sides to achieve better coverage. While more LEDs per wavelength could give better coverage, the USB supply voltage limits the number of LEDs per string for safe operations (LED forward voltage is about 1.5 V, LED driver dropout voltage is a few hundred mV, and USB voltage can drop to 4.5 V). For more LEDs, some kind of boost regulation or external power supply is necessary. As all LEDs are driven by the same current, the difference in output power is equalized with the software-based neutral calibration (white point correction).

The employed LED drivers are standard components from automotive or (background) lighting applications and are available in linear or switching variants. They can drive LEDs with high accuracy (e.g., temperature stability) and usually have a dimming pin that can be directly controlled by a PWM signal. It must be considered that these components often have asymmetrical propagation delay/transition behavior (time to turn on and off). When using higher PWM frequencies, this could lead to a slight distortion in the received signal (e.g., addition of even harmonics). Sometimes, this can be compensated by adjusting the PWM duty cycle so that the receiver receives a rectangular wave again.

The transmitter hosts a ToF sensor to extend the remission measurement with additional distance information. While the structure of the proposed multispectral sensor is very similar to continuous-wave time-of-flight, the phase-shift information cannot be used to calculate the measurement distance. For this, special high-speed optoelectronics would have to be used (e.g., laser diodes and avalanche photodiodes) to be able to use carrier frequencies of several MHz. Instead, we use a low-cost consumer-grade ToF sensor to gain (nearly) the same functionality. With this additional information, especially during standalone operation, the presence of measurement objects and their correct distance can be detected. Thus, invalid measurements outside the intended measurement range can easily be identified and discarded.

### 3.4. Optical Shielding

The optical shielding is responsible for removing any unwanted direct radiation transmission between the transmitter and receiver. Ideally, there should be no detectable optical signal when nothing is in front of the sensor system. Practically, there are always unwanted internal and external reflections that can disturb proper sensor operations. Furthermore, some materials are transparent in the near-infrared while opaque in the visible (and vice versa). For example, the sensor aperture is covered with an acrylic protection screen that looks black-tinted in the visible (often used to hide infrared-based systems). Also, many (3D-printed) materials and PCBs are not fully opaque in the NIR, which must be taken into account.

**Figure 6:**
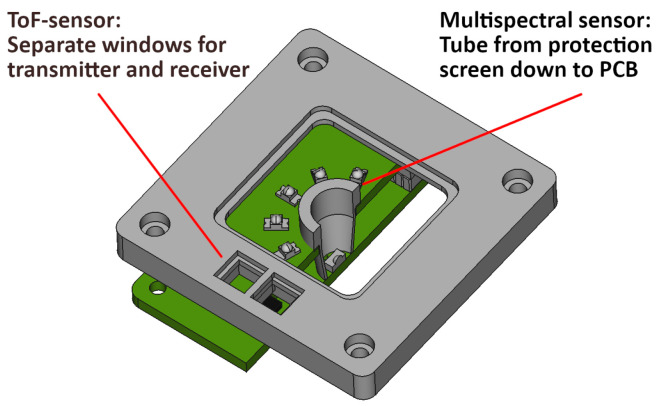
The optical shielding of the proposed sensor.

The optical shielding of the proposed sensor is illustrated in Figure 6 and consists of two separate parts:

**Multispectral sensor:**
  Despite focusing, the NIR LEDs emit small amounts of light in virtually any direction. To isolate the photodiode, a small tube is used that extends from the protective shield (above the LEDs) to the receiver board. The 3D-printed part is coated with a near-infrared-absorbing paint to suppress any remaining internal reflections. In the focused receiver variant, the tube can also be used as a lens holder at the same time.
**ToF sensor:**
  The transmitter and receiver should not be placed behind the same (protective) screen, as even small reflections could disable the sensitive time-detection circuits. The manufacturer’s recommendations are integrated into the top cover, consisting of two small windows with a fillet in between that extends to the ToF sensor and separates the two parts.

Each sensor design may require its own optical shielding solution. In addition, external influences (e.g., additional protective screens/windows) that are not within the developer’s control can often affect the proper functioning of the sensor. Therefore, the current optical shielding must be reviewed and, if necessary, revised when integrating with other systems.

## 4. Software Design Considerations

The software development for the proposed sensor system is divided into embedded firmware and an optional desktop application. While the embedded controller firmware is responsible for the correct acquisition of measurement data, the desktop counterpart is designed to support the user in carrying out experiments. As already mentioned, the signal processing is completely digital, and the analog filter is only needed for signal preparation before digitization. More detailed information about the digital signal processing can be found in Section 4.1.

The embedded firmware uses middle-layer software provided by the manufacturer (e.g., USB middleware) to shorten the initial development time. Since most of the USB stack is executed in software, and communication via USB can be a known bottleneck, we use DMA transfers (Direct Memory Access) at every opportunity to reduce the CPU load and achieve the maximum possible transfer rates. For demonstration purposes, the controller is able to process the measurements directly without an external desktop computer. The (binary) classification result can then be outputted via the RGB-LED or a general-purpose input/output (GPIO) on the extension header. Since embedded software should ideally not use dynamic memory allocation, we used a fixed content size implementation for the FFT. For this purpose, the window size (the number of samples in a measurement) must be known at the compile time and cannot be changed at runtime. The controller should be accessible via the standard JTAG debug interface when fully assembled. This should enable in-system debugging and programming to ensure fast and secure firmware development without additional difficulties (e.g., incorrect assembly and worn contacts).

Although the sensor system could also run without additional desktop software running on a PC, it was decided to develop a useful, ready-to-use application that covers most of the functions that may be encountered in practice. The developed desktop application for the proposed sensor system has the following features:

**Sensor status:**
  A display of system health status that includes some additional sensor information (e.g., power consumption and ambient temperature), as well as some software-defined error flags (e.g., internal communication bus error). This provides a quick overview of the current operating status of the sensor.
**Parameter control:**
  The control of many system parameters at runtime, enabling a wide range of experiments. All measurement-acquisition-related parameters (e.g., carrier frequencies or sample rates) can be saved in the persistent flash memory of the microcontroller to be available at the next startup. Other settings (e.g., calibrations and classifiers) are stored in an organized configuration file, and mapping with the corresponding sensor specimen is performed via a unique identifier provided by the microcontroller.
**Measurement visualization:**
  The visualization of raw measurement data (time/frequency domain) and classification results for a fast on-site analysis. The data can either be live-captured or loaded from a disk.
**Measurement handling:**
  The collection of measurements with optional meta-information (e.g., current material) for later re-visualization or processing. With built-in methods, the measurement collection can be used to generate calibration data or for classifier training procedures. Also, simple file operations (save, load, export to CSV) are supported for further processing or analysis with other software (e.g., data mining).

### 4.1. Digital Signal Processing

The complete digital signal processing chain consists of four sequential steps, and the (digital) lock-in amplifier is only one part of it. The input is usually a measurement window (array) of a-few-milliseconds signal acquisition, acquired at a few hundred thousand samples per second. This leads to a response time of several hundred classification results per second, which is fast enough for most safety and security applications. Each step in the chain has the following task:

**Preprocessing:**
  Obtaining the uncalibrated remission values of the target surface is the first step in the digital signal processing chain. Since the modulation frequencies of the carrier signals are known, the relevant magnitudes can be extracted with known methods (i.e., only the fundamental frequency, ignore the remaining harmonics from the rectangular wave form). A lock-in approach (precalculated sine/cosine tables) is used when only few magnitudes are needed and computation power is of concern. Otherwise, with an FFT, a more extensive signal analysis is possible to discard problematic measurements (e.g., scan for leakages indicating unwanted interference).
**Calibration:**
  A basic calibration consists of a dark offset subtraction and multiplicative white point correction. The dark offset must be determined with full lighting running and no direct optical reflection from the transmitter to the receiver. Only in this way can the remaining electrical and optical crosstalk be recorded. The white calibration should be performed with a long-term stable material with even reflectivity in the near-infrared spectrum. Since professional laboratory white references are often short-lived, we use other inexpensive materials like Polytetrafluoroethylene (PTFE) as a white reference replacement (more rigid and sufficiently evenly distributed in targeted near-infrared spectral range). With a white calibration, the differences between specimens of the same sensor type due to part variances are compensated. Only with a properly functioning calibration are the classification settings transferable between the sensor systems.
**Feature extraction:**
  The calibrated remission values r(λ) are still dependent on the distance between the target surface and the receiver. An often-employed feature-space transformation is the normalized differences $nd(\lambda_{a},\lambda_{b})$, the difference between two signal values r(λ) derived at wavelengths $\lambda_{a}$ and $\lambda_{b}$ divided by their sum:
  (1) $$nd\left(\lambda_{a},\lambda_{b}\right)=\frac{r\left(\lambda_{a}\right)-r\left(\lambda_{b}\right)}{r\left(\lambda_{a}\right)+r\left(\lambda_{b}\right)}$$
  The value range of the normalized differences is always between −1 and 1. From the five remission values at different wavelengths, a total of 10 differences for further material identification can be calculated.
**Material classification:**
  As previously mentioned, complex machine-learning-based classification is usually not required here. In many cases, a simple threshold over the relevant differences is already sufficient for the targeted application. More sophisticated approaches with the use of statistical methods can provide certain pseudo-probabilities for a known material, which can provide more insight than a (binary) classification result.

## 5. First Experimental Results

**Figure 7:**
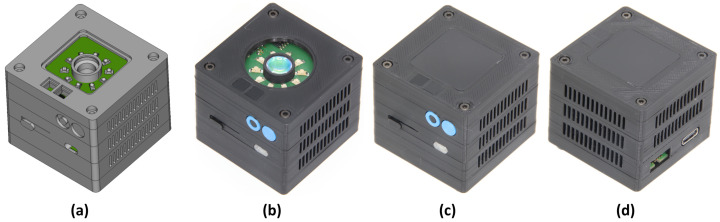
Sensor images: (**a**) Closed Variant Front (CAD). (**b**) Focused Variant (Photo). (**c**,**d**) Closed Variant Front and Back (Photo)).

Several sensor specimens were built for further experiments. As described in Section 3.2, two different variants were produced, which are shown in Figure 7. The 3D-printed enclosure has compact dimensions (40 mm × 40 mm × 35 mm — L × W × H). Ventilation slots for cooling were added to aid passive heat dissipation. The top cover is closed with a black-tinted acrylic protection screen, which is transparent in the near-infrared.

### 5.1. General Operation

The sensor was designed for near-field usage and shows good signal strength up to a 30 cm measurement distance. At larger distances, the 1550 nm signal is no longer detectable with some materials (e.g., skin has a strong absorbance of this wavelength), which directly influences the differences and material classification. Thus, to achieve greater measurement ranges, a more sophisticated transmitter and a more focused receiver may be necessary.

The first runtime tests showed a current consumption of 130 mA in standby mode and 270 mA during measurement, with about 100 mA of this being consumed by the isolation (load-independent). The supply voltage needs to be at least 4.5 V for the proper function of the analog circuitry. While the voltage and current requirements are within USB specifications, it has been shown that a weak power supply or a bad USB cable can result in a high voltage drop. It must be considered that with rising loads, the voltage drop can be further intensified. Fortunately, this can be easily detected by the power monitor so that the user is warned when the supply voltage drops too low.

**Figure 8:**
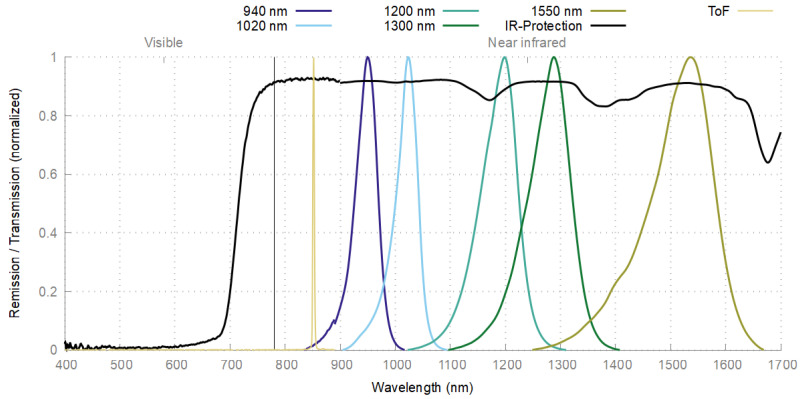
LED remission/NIR-protection transmission spectra.

For control purposes, the sensor was also measured spectrometrically. Figure 8 shows the remission spectra of all NIR-LEDs and the ToF sensor, as well as the transmission spectrum of the protection screen. As expected, the spectral bandwidth of the NIR-LEDs is rather wide and increases with higher wavelengths. In contrast, the ToF sensor has a narrow bandwidth, since it uses laser lighting with a wavelength of about 940 nm. The protective screen has its passband above 700 nm and is thus transparent to all wavelengths used in the proposed sensor system.

### 5.2. Surface Material Classification

Material classification is based on normalized differences, as described in Section 4.1, and depending on which materials are to be detected, a given difference may contribute more or less to the final result. By inspecting the numerical values of the differences for each material class separately, it should easily be observable if a specific difference is suitable for a material classification decision.

**Figure 9:**
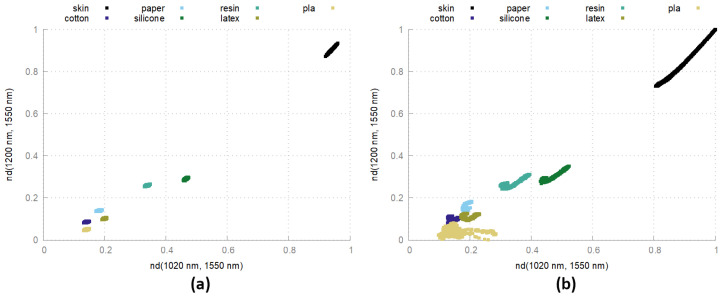
Normalized differences of human skin and exemplary artifact materials. (**a**) Fixed distance at 20 cm. (**b**) Full range from 5 cm to 30 cm.

Figure 9 shows two types of differences in some material measurements (same materials as in Figure 1) at a fixed measurement distance and their full measurement range. In this visualization, the material classes form distinct point clouds and can easily be separated from each other. Since the chosen differences are especially suited to distinguish skin from many other materials, the skin point cloud is far away from the other observed materials. As can be easily seen, separation is possible with a simple threshold.

When observing the measurements at different measurement distances, a shift in the differences can be recognized, which results in stretched point clouds in Figure 9. A reason for this could be the simple lighting configuration and could be mitigated by extending the calibration by a distance-based correction.

The detailed evaluation of the PAD sensor on the hand palms and faces of more than 300 test candidates is still ongoing and will be reported in further publications.

## 6. Conclusions and Outlook

In this work, we have shown that with only off-the-shelf components and reasonable effort, a multispectral sensor system can be built for reliable surface material classification that is well suited for PAD in multiple biometric applications. The sensor is largely independent of ambient light and human skin type and is currently being evaluated in real applications at our biometric evaluation center.

Although the proposed sensor system already works well for the targeted applications, there are still opportunities for future improvements. The following aspects of the hardware and software could be further improved:

**Extension of calibration:**
  The currently used calibration only compensates the different output powers for each wavelength (white) and the remaining background noise (dark). Adding more calibration components could further enhance the system’s precision in difficult situations. As shown in Figure 9, there is a distance-dependent drift in the differences that is likely due to the non-ideal lighting configuration. Also, the carrier signals are attenuated differently for each chosen modulation frequency, since the analog parts of both the transmitter and receiver introduce frequency-dependent behavior. A frequency-based compensation would allow for changing carrier frequencies, which could make the sensor more resilient to attacks and more adaptable to its environment.
**Enhancement of classification algorithms:**
  In this work, we have shown that a simple threshold for normalized differences can often be used to distinguish between specific materials. More recent approaches from the authors use a statistical classification approach, which can be trained on a specific material and can output a form of pseudo-probability for new measurements. When combining several of these, a simple multi-classification algorithm should be possible that can output the best matches for all previously learned materials.
**Laser-based lighting configuration:**
  The previously developed near-infrared sensor systems used an LED-based lighting configuration because it was readily available, inexpensive and safe to use in most situations. While this worked well for most targeted applications, only rather coarse spectral details can be seen with (wideband) LEDs. A solution could be the use of laser-based lighting to detect smallband details, potentially enhancing the performance for specific materials and applications.
**Single-pixel camera:**
  There are already different concepts on how to take a complete image with a single photodiode. All of these include some micro-electromechanical systems (MEMSs) to control either the outgoing (e.g., scan area with laser beam [16]) or incoming light (e.g., compressed sensing [17]). Since InGaAs-based near-infrared image sensors are quite expensive, the reduction to a single photodiode could be worth the effort and could enable a wide range of new applications.
**Consumer-grade SWIR imaging:**
  In the past five years, there has been a noticeable increase in technical reports and publications focusing on short-wavelength infrared (SWIR) image sensors, specifically in the wavelength range of 1000 to 2000 nm (e.g., quantum dot [18,19] or coating [20,21]). Historically, the primary technology for sensors in this range has been based on InGaAs, which often entails costs reaching tens of thousands of US dollars. Even though emerging technologies promise cost reductions by a factor of 2 to 3, they still represent a significant financial barrier to integration into most product classes. This cost issue is a primary reason why there is growing interest in point detectors. The algorithmic approaches and application areas for these detectors are expected to be easily transferable to 2D sensor technology in the future once more cost-effective solutions become available.

## Figures and Tables

**Table 1:** NIR-LEDs of the proposed sensor.

Type	$\lambda_{p}$ (nm)	$\Delta \lambda_{0.5}$ (nm)	$\Phi_{e}$ (mW)	φ (°)
EOLS-940-843	940	30	13.5	40
EOLS-1020-843	1020	40	10	40
EOLS-1200-844	1200	73	12	40
EOLS-1300-844	1300	78	11	40
EOLS-1550-844	1550	120	6	40

## Data Availability

The raw data supporting the conclusions of this article will be made available by the authors on request.
